# IL-4 modified expanded polytetrafluoroethylene (e-PTFE) surgical patch promotes angiogenesis in transplanted flap and inhibits inflammatory response

**DOI:** 10.1186/s12893-023-02024-4

**Published:** 2023-05-27

**Authors:** Peng Song, Yizheng Liu, Chenfei Du, Zhen Lei, Jinwei Ai, Guanghui Li, Kai Jing

**Affiliations:** 1 Department of Burns Microsurgery, Henan Province Hospital of TCM, the Second Affiliated Hospital of Henan University of Chinese Medicine, No. 6 Dongfeng Road, Jinshui District, Zhengzhou, Henan Province 450000 China; 2 The Central Laboratory, Henan Province Hospital of TCM, the Second Affiliated Hospital of Henan University of Chinese Medicine, Zhengzhou, China

**Keywords:** Expended polytetrafluoroethylene, IL-4, Skin flap transplantation, Angiogenesis

## Abstract

Skin flap transplantation is one of the most common tissue transplantation methods for wound repair and organ reconstruction in plastic surgery. During the transplantation process, the inflammatory response of transplanted flap and angiogenesis are critical to the successful rate of skin flap transplantation. In recent years, to improve the biocompatibility and cell affinity of biomedical materials, the modified biomaterials have gradually become a popular subject in scientific researches. In our study, the IL-4 modified expanded polytetrafluoroethylene (e-PTFE) surgical patch IL4-e-PTFE was prepared, and the rat skin flap transplantation model was constructed. The results of cell experiment prove that IL-4 has potentiation in the angiogenesis of human umbilical vein endothelial cell (HUVEC) induced by monocyte, and IL-4 can also promote angiogenesis by inducing the M2 macrophages. According to the results of in vivo experiment, the apoptosis level of transplanted flap cells of rats in the IL4-e-PTFE group was lower than that in the e-PTFE group, and in the IL4-e-PTFE group, the expression levels of pro-inflammatory cytokines IL-1β, IL-6 and TNF-α showed significantly decline compared to the e-PTFE group, while the expression levels of anti-inflammatory cytokines IL-1Ra, IL-10 and TGF-β presented significant increase compared to the e-PTFE group; the immunofluorescence staining results show that the number of M2 macrophages in transplanted flap area of rats in the IL4-e-PTFE group was significantly higher than that in the e-PTFE group, and the angiogenesis level was remarkably improved. In this study, by preparing IL4-e-PTFE and carrying out the cell and in vivo experiments, a reference method is proposed, which can reduce the inflammatory response during skin transplantation process using e-PTFE and optimize the long-term effects of flap blood vessels, hoping to provide a broader space for the applications of e-PTFE in medicine.

**Supplementary Information:**

The online version contains supplementary material available at 10.1186/s12893-023-02024-4.

## Introduction

Expended polytetrafluoroethylene (e-PTFE) is a high polymer material for medical use, and due to its characteristics of inertia, microporous structure and corrosion resistance[1], it has broad applications in biomedical engineering. For example, the membrane tube implant made of e-PTFE membrane and silicone tube can be used to treat refractory glaucoma[2]; the covered stent covered by e-PTFE cab effectively alleviate the refractory ascites of liver cirrhosis patients[3]; the e-PTFE flake stent anchored with rigid nylon foil can be used in the reconstruction of fornical conjunctiva[4]; the e-PTFE soft tissue patch and vascularized flap can be used in the chest wall reconstruction of patients who had chest wall resection[5]. With more clinical applications, e-PTFE has gradually replaced the traditional silicone rubber and become one of the most ideal biological tissue substitutes.

During the process of skin transplantation, skin flap transplantation is one of the most common tissue transplantation methods for wound repair and organ reconstruction in plastic surgery[6]. The survival of skin flap depends on sufficient blood supply, and blood is delivered via the blood vessel ganglions of skin flap pedicle[7]. However, blood supply gradually decreases along the skin flap toward the distal part[8], which often leads to necrosis of the distal part of skin flap due to ischemia[9]. Furthermore, inflammation plays an important role in the survival of skin flap. When there is inflammatory response, moderate coagulative necrosis can be detected on the epidermis of skin flap, associated with inflammatory cell infiltration, and the more severe the necrosis is, the more obvious the inflammation[10]. These factors have seriously affected the clinical application of skin flap transplantation. This indicates that during the process of skin transplantation, the inflammatory response of transplanted flap and angiogenesis are critical to the successful rate of skin flap transplantation.

Interleukin-4 (IL-4) is a pleiotropic cytokine, which can regulate polarization of macrophages to the alternatively activated (M2) phenotype[11]. Under stress conditions, the macrophages are polarized, and the polarized macrophage subtypes (M1/M2) can delicately regulate and respond to various different stimulations, which play a critical role in inflammatory response. It is reported that the M1 macrophage has pro-inflammatory effect, while the M2 macrophage can promote inflammation resolution via anti-inflammatory cytokines, so as to inactivate the pro-inflammatory cytokine phenotype and reconstruct homeostasis[12]. This indicates that IL-4 has anti-inflammatory effect. Moreover, IL-4 can also promote angiogenesis. For instance, Tian et al[13]. reported that IL-4 can also promote angiogenesis by secreting exosome containing miRNA-26a. Matthew Craig et al. proved that significant increase of several angiogenesis factors can be achieved by using IL-4 to stimulate the U937 cells[14].

In recent years, to improve the biocompatibility and cell affinity of biomedical materials, people have started to study modification of biomaterials. For example, hydroxyapatite is an implant material, which does not support cell attachment itself, but after adsorbing biomimetic peptide Glu7-Pro-Arg-Gly-Asp-Thr rich in acidic amino acid Glu onto the surface of hydroxyapatite, it can promote the attachment and expansion of bone cells[15]. The titanium implant with insulin coating can promote the bone formation of animals with osteoporosis[16]. With the development of modern medical technologies, the applications of e-PTFE in the biomedical field are increasing. However, the influence of IL-4 modified e-PTFE surgical patch in the process of skin flap transplantation is still unclear, and the internal mechanism of generating corresponding influence still needs to be found. Here, we carry out related study, hoping to provide a broader space for the applications of e-PTFE in the biomedical field. We present the following article in accordance with the ARRIVE reporting checklist.

## Materials and methods

### Preparation of IL-4 modified e-PTFE (IL4-e-PTFE) surgical patch

By referring to the study of Assmann[17, 18], we used 172 nm UV light to irradiate on PTFE in the ammonia environment to change its polarization, so as to obtain e-PTFE with single polarization change. The recombinant human-rat IL-4 protein was dissolved in in the PBS containing 0.1% BD Matrigel to prepare the incubation buffer. The e-PTFE with single polarization change was added to the incubation buffer and incubated under 4℃ overnight, so as to ensure the IL-4 modification was in the polarization area of e-PTFE. Then, it was washed twice using PBS to remove free IL-4. After drying under ventilation, ethylene oxide was used for sterilization, and the IL4-e-PTFE surgical patch was prepared for subsequent animal experiment.

### Separation and culture of macrophage

The 8–10 weeks’ old male SD rats were bought from Vital River (certificate No.: 1,100,111,911,045,302), they were bred in the Laboratory Animal Center of Henan Province Hospital of TCM [SYXK (Yu) 2016-0009], and the animal ethics certificate number was 20180316WZ. The study was carried out by strictly following The Guide for Care and Use of Laboratory Animals formulated by National Institutes of Health (NIH). All experiments were performed in accordance with ARRIVE guidelines. This study was approved by the Ethics Committee of Henan Province Hospital of TCM. The rat was killed via cervical dislocation, soaked in 75% ethanol for 5 min, and then place on the dissecting table. The enterocoelia of rat was opened under sterile conditions, and intraperitoneal injection of RPMI-1640 culture solution containing no serum was conducted. The abdomen was gently rubbed for 5 min and let stand for 6 min, a syringe was used to extract 4mL peritoneal fluid and put into a frozen centrifuge tube, and this process was repeated twice. The TrisNH4Cl solution was used to dissolve erythrocytes in peritoneal fluid, and then the cells were washed. Finally, the specimen was added to the RPMI-1640 medium containing 10% fetal calf serum to re-suspend cells and adjust the cell concentration to 2 × 10^6^mL-1. Next, the specimen was inoculated to a 50mL culture flask for culture under the conditions of 37℃ and 5% CO_2_ until the logarithmic phase. Next, the macrophages of logarithmic phase were inoculated to 6-well plates according to the concentration of 2 × 10^6^ mL^− 1^, and the control group and IL-4 group were set, three wells for each group. After the cells were cultured to grow to the 80% fusion state, IL-4 was added to the IL-4 group to reach the final concentration of 10 ng/ml, and the control group had no treatment. Then, after continuous culture of 12 h, cells were collected for subsequent experiment.

### Angiogenesis experiment

The human umbilical vein endothelial cells (HUVECs) were bought from Life Technologies (Gibco; C-003-5 C) and saved in the Central Laboratory of Henan Province Hospital of TCM, and monocytes were separated from rats according to the method of Chometon[19]. Based on the experimental design, the rats were divided into four groups in the angiogenesis experiment, which were the control group (NC), IL-4 group, monocyte group and IL4 + monocyte group, respectively, three wells for each group. First, 10 ul Matrigel was added to each well of angiogenesis slide; after matrigel coagulated, 50 ul HUVEC cell suspension at logarithmic phase was added to each well, and the cell number was 1 × 10^4^. After the cells grew to the 80% fusion state, the cells were treated correspondingly. No treatment was provided to the control group; for the IL-4 group, IL-4 was added to reach the final concentration of 10 ng/ml; in the monocyte group, monocytes were added in the concentration of 1 × 10^6^ mL^− 1^, and then, they were co-cultured with HUVEC; in the IL4 + monocyte group, in addition to adding IL-4 to reach the final concentration of 10 ng/ml, monocytes were also added in the concentration of 1 × 10^6^ mL^− 1^ for co-culture with HUVEC. Finally, the specimens were cultured in the incubator under the conditions of 37℃ and 5% CO_2_ for 16 h, and then, the phase contrast microscope was used to observe the angiogenesis situation.

### Skin transplantation model

The rats were randomly divided into the e-PTFE group (n = 18) and IL4-e-PTFE group (n = 18). Intraperitoneal injection of pentobarbital sodium (40 mg/kg) was conducted to anesthetize the rats. Then, the “McFarlene Flap”[20] model was used to separate a 1 cm × 2 cm skin myocutaneous flap based on tail from the lower fascia on the rat’s back, and measures were taken to ensure two arteries supporting blood supply of this skin flap model were completely cut off. After hemostasis, for the IL4-e-PTFE group, the e-PTFE membrane was spread on the inner surface of skin flap, and after ensuring the entire flap was covered by the e-PTFE membrane, the skin flap was sutured at the wound. In the e-PTFE group, the e-PTFE membrane was spread on the inner surface of skin flap, and then sutured. Rats of these two groups were bred in standard experimental cages, and standard rat food and water were provided. Seven days later, all rats were injected with excessive pentobarbital sodium for euthanasia, and immunohistochemistry and Western blot analyses were carried out.

### Immunohistochemistry analysis

The skin and flap tissue specimen of rats were collected. All skin flap specimens were fixed using 10% formalin. The skin flap was cut into sections and embedded in wax, and then, the hematoxylin-eosin (HE) was used for staining. After dewaxing and hydration of sections, hydrogen peroxide was used to mount endogenous peroxidase and citrate buffer for antigen retrieval, then, it was added with PBS containing 5% Bovine Serum Albumin (BSA) and 1% Tween-20 for mounting, and primary antibody was added under 4℃ for overnight (phosphate buffer was used to replace primary antibody as negative control); after washing with PBS, the IgG secondary antibody with HRP marker was added, and the specimen was incubated at room temperature for 30 min; after washing, the DAB chromogenic agent was used for chromogenic reaction; finally, hematoxylin was used for redyeing, and then differentiation, dehydration, vitrification and mounting were carried out successively.

### Real-time quantitative polymerasechain reaction

RT-qPCR was carried out to check the expression levels of CD31, CD36 and PDEC-GF mRNA in tissues. The main process is as follows: Trizol (Sigma, USA) was used to separate total RNA from the cell, and the Prime Script RT lit (Takara, Japan) was used to synthesize cDNA. Table 1 lists the primer sequences used for RT-qPCR. In the Bio-Rad iQ5 fluorescence ration system (BIO-RAD, USA), Power SYBR Green PCR Master MIX (ABI, USA) was used to carry out real-time ration RT-PCR on the 96-well plate according to the specifications and steps in the manual, so as to analyze the expression levels of corresponding genes.

**Table 1:** Primer sequences of RT-PCR

Primer	Sequences
CD31-qF	TCCAGAAGAACTCCAATG
CD31-qR	GGCAATTATCCACTATACAC
CD36-qF	CATAGGACATACTTGGAT
CD36-qR	TCTCTTCAGATTCTTCAG
PD-ECGF-qF	AGGACCAGGACTATATTGTG
PD-ECGF-qR	AGTAATCAGTGAAGAGACCAT
β-Actin-qF	CTAAGGCCAACCGTGAAAAG
β-Actin-qR	AACACAGCCTGGATGGCTA

### Western blot

The skin flap specimens were collected and placed in the RIPA lysate (Sigma, USA) containing protease inhibitor. After lysis on ice for 30 min, the filtrate was collected and stored under − 20℃. The BCA Protein Quantification Kit (Vazyme, China) was used to measure the protein concentration. After electrophoretic separation of equivalent proteins on 10% SDS-PAGE gel, the proteins were blotted to the PVDF membrane. After transferring membrane, the specimen was mounted at room temperature for 2 h, then it was added with primary antibody for incubation under 4℃ overnight, in the next step, secondary antibody was added for incubation for 2 h, and finally, the chromogenic agent was added for exposure.

### Detection of apoptotic cells using TUNEL staining method

The wax sections were dewaxed and hydrated and incubated for 30 min in the protease K working solution under 37℃; then, the TUNEL reaction mixture was added to incubate for 60 min in a cassette under 37℃; the transformational POD signals were used to transform for 30 min, and the DAB chromogenic agent reacted with substrate to show color; next, hematoxylin was used to dye the cell nucleus, and then, the specimen was dehydrated, vitrified using xylene, and mounted with neutral balsam. The apoptotic cells in transplanted flaps in different groups were counted under an optical microscope, and pictures were taken.

### Detection of inflammatory factors in serum using the ELISA method

After setting and centrifugalization of blood collected from rats in various groups, the serum was stored under − 80℃. After the reagent and serum samples were balanced to room temperature, operation was conducted according to the test steps in the manual of ELISA kit (Invitrogen, USA). The ELISA instrument was used to carry out 450 nm wavelength detection, and the OD value of sample was calculated according to the standard curve.

### Statistical analysis

All data was expressed as mean ± SD. Software GraphPad Prism 8 was used for data analysis. One-Way ANOVA and least significant difference (LSD) were employed to detect the difference between two groups. When P<0.05, the difference is considered statistically significant.

## Results

### IL-4 plus monocyte promote angiogenesis

**Fig. 1:**
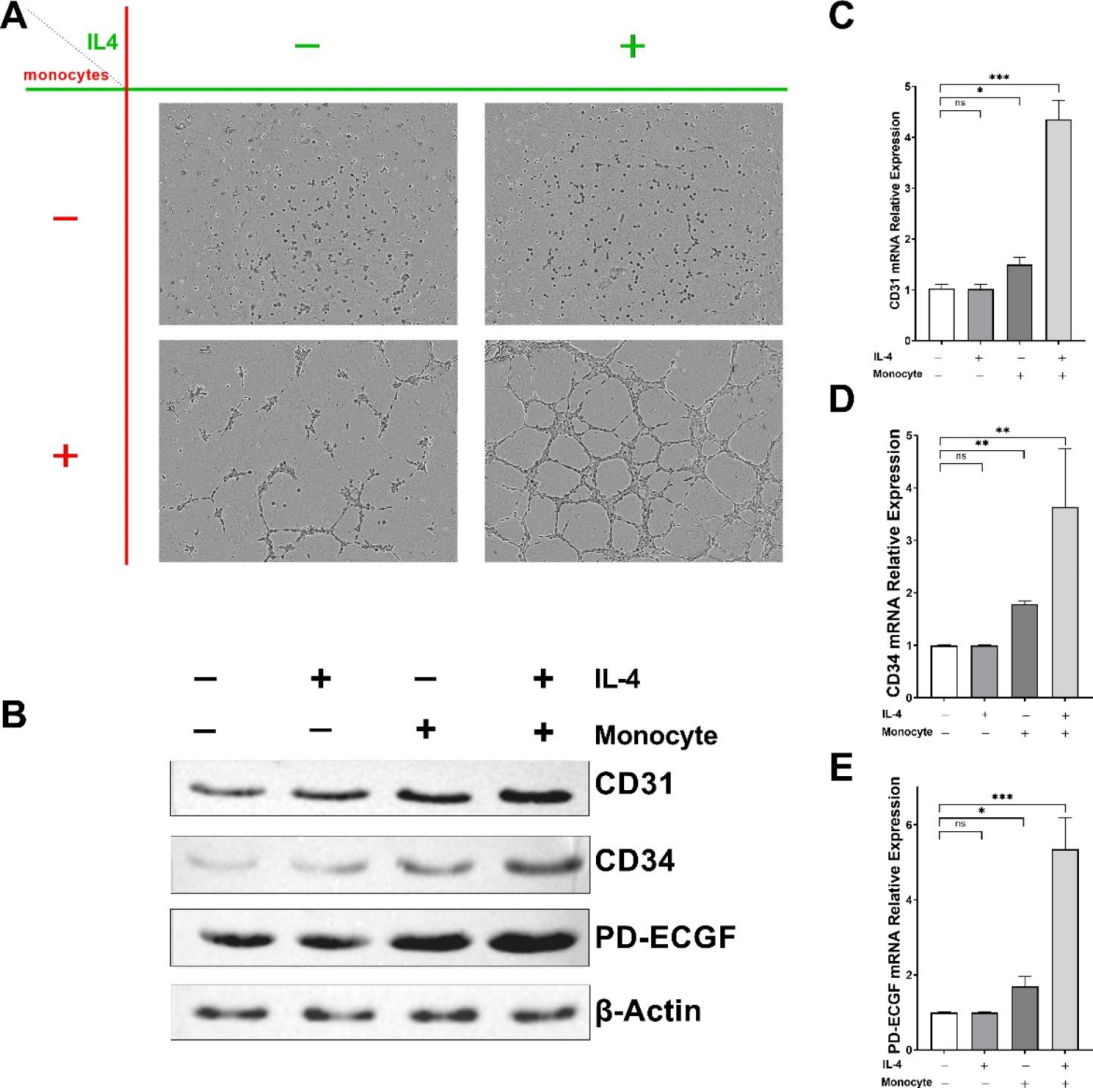
IL-4 promotes HUVEC to form new vessels. **A**: HUVEC angiogenesis situation in different groups under phase contrast microscope; **B**: Using WB to detect the protein expressions of vascular endothelial cell markers; **C**: Relative expression of CD31 mRNA; **D**: Relative expression of CD36 mRNA; **E**: Relative expression of PDEC-GF mRNA

We checked the HUVEC angiogenesis situation with different treatments. As shown in Fig. 1A, under the phase contrast microscope, the IL-4 group did not present significant difference from the control group in terms of angiogenesis; on the other hand, both the monocyte group and IL4 + monocyte group had angiogenesis, and the angiogenesis of IL4 + monocyte group was more obvious. Later, we carried out the WB experiment to detect the protein expressions of vascular endothelial cell markers and vascular endothelial growth factor (VEGF), and the results are presented in Fig. 1B. Compared to the control group and IL-4 group, the expressions of vascular endothelial cell markers CD31 and CD34 and the vascular endothelial growth factor PDEC-GF of both the monocyte group and IL4 + monocyte group increased, and the increase of the IL4 + monocyte group was more obvious. Then, we tested the transcription levels of the markers mentioned above via qPCR, and the results showed consistent trend with WB. The statistical results are shown in Fig. 1C-E. The above results indicate that IL-4 has potentiation effect in HUVEC angiogenesis induced by monocyte.

### IL-4 promotes angiogenesis by inducing M2 macrophages

**Fig. 2:**
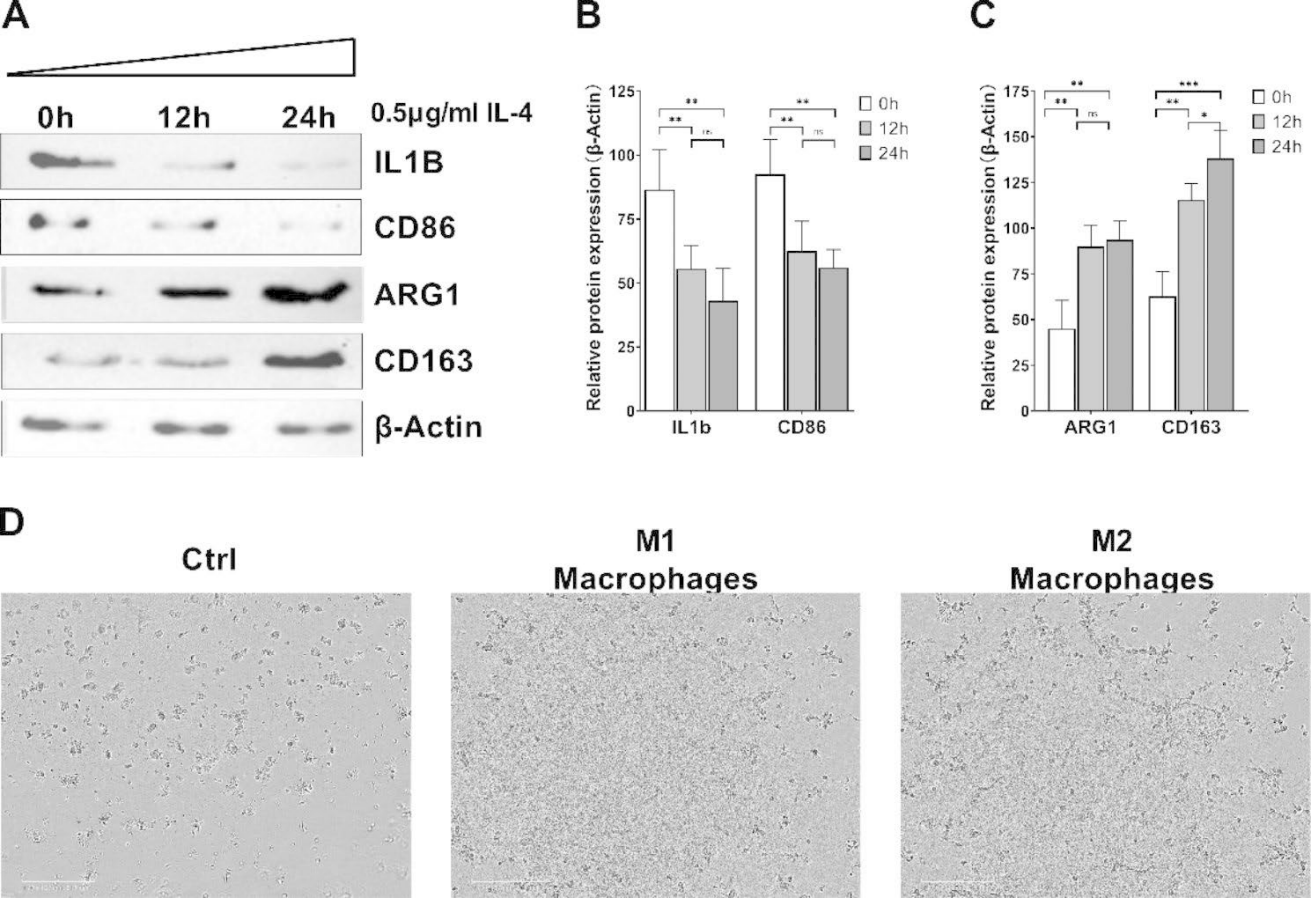
IL-4 promotes HUVEC angiogenesis by inducing M2 macrophages. **A**: Using WB to detect the protein expressions of M1 and M2 macrophages; **B**: Relative protein expressions of M1 macrophage markers; **C**. Relative protein expressions of M2 macrophage markers; **D**: Observation of HUVEC angiogenesis in different groups using phase contrast microscope

Considering that IL-4 has potentiation effect in HUVEC angiogenesis induced by monocyte, we carried out IL-4 treatment of macrophages cultured in vitro and detected the cell markers via WB, and the results are shown in Fig. 2A-C. With the extension of time in IL-4 treatment, the expressions of M1 macrophage markers IL1B and CD86 significantly reduced, while the expressions of M2 macrophage markers showed remarkable increase. After co-culture of M1 macrophages and M2 macrophages with HUVEC, the phase contrast microscope was used to observe angiogenesis. The results are presented in Fig. 2D. Compared to the control group, the group with M2 macrophage treatment showed increase in HUVEC angiogenesis. The above results prove that IL-4 can promote angiogenesis by inducing M2 macrophages.

### IL4-e-PTFE inhibits apoptosis and inflammatory response in body

**Fig. 3:**
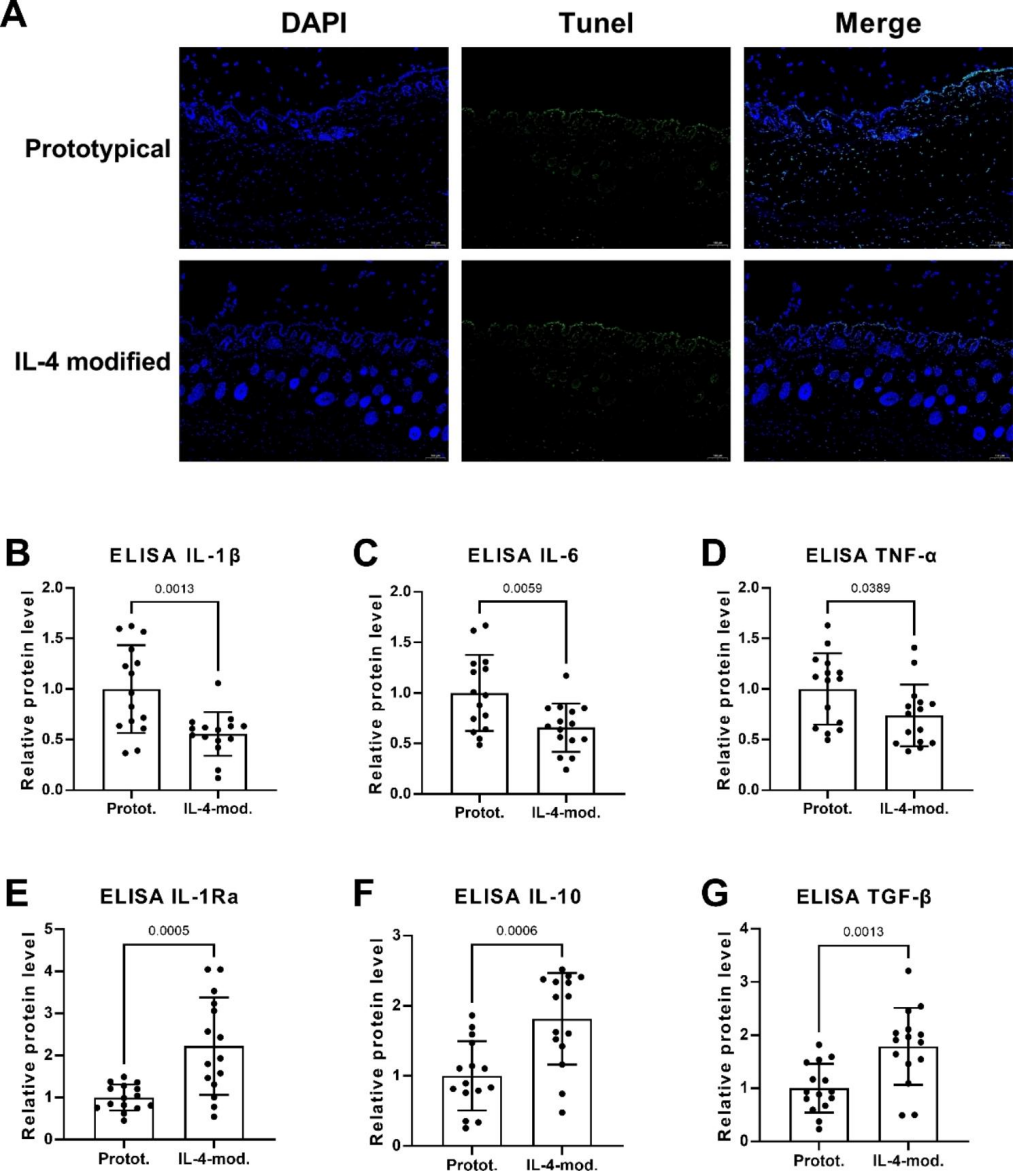
IL4-e-PTFE inhibits in vivo apoptosis and inflammatory response. **A**: Using Tunel to detect the apoptosis of transplanted flaps in different groups; **B**-**G**: Using ELISA to detect the in vivo inflammatory factor levels of rats with skin flap transplantation in different groups

Then, we used e-PTFE and IL4-e-PTFE in skin flap transplantation of rats, respectively, and tested the apoptosis levels of transplanted flaps in different groups. The Tunel results are shown in Fig. 3A. The green fluorescence level of cells in the IL4-e-PTFE group was significantly lower than that in the e-PTFE group, which means that the IL4-e-PTFE group had lower apoptosis level than the e-PTFE group. This result indicates that the IL4 modification can inhibit apoptosis in the transplanted flap to a certain extent. ELISA was used to detect the inflammatory factor level of rats with skin flap transplantation, and the results are presented in Fig. 3B-G. In the IL4-e-PTFE group, the expression levels of pro-inflammatory cytokines IL-1β, IL-6 and TNF-α showed significant decline compared to the e-PTFE group, while the expression levels of anti-inflammatory cytokines IL-1Ra, IL-10 and TGF-β presented remarkable increase compared to the e-PTFE group. In other words, IL-4-e-PTF can reduce inflammatory response of rats with skin flap transplantation.

### IL4-e-PTFE promotes angiogenesis of skin flap in vivo

**Fig. 4:**
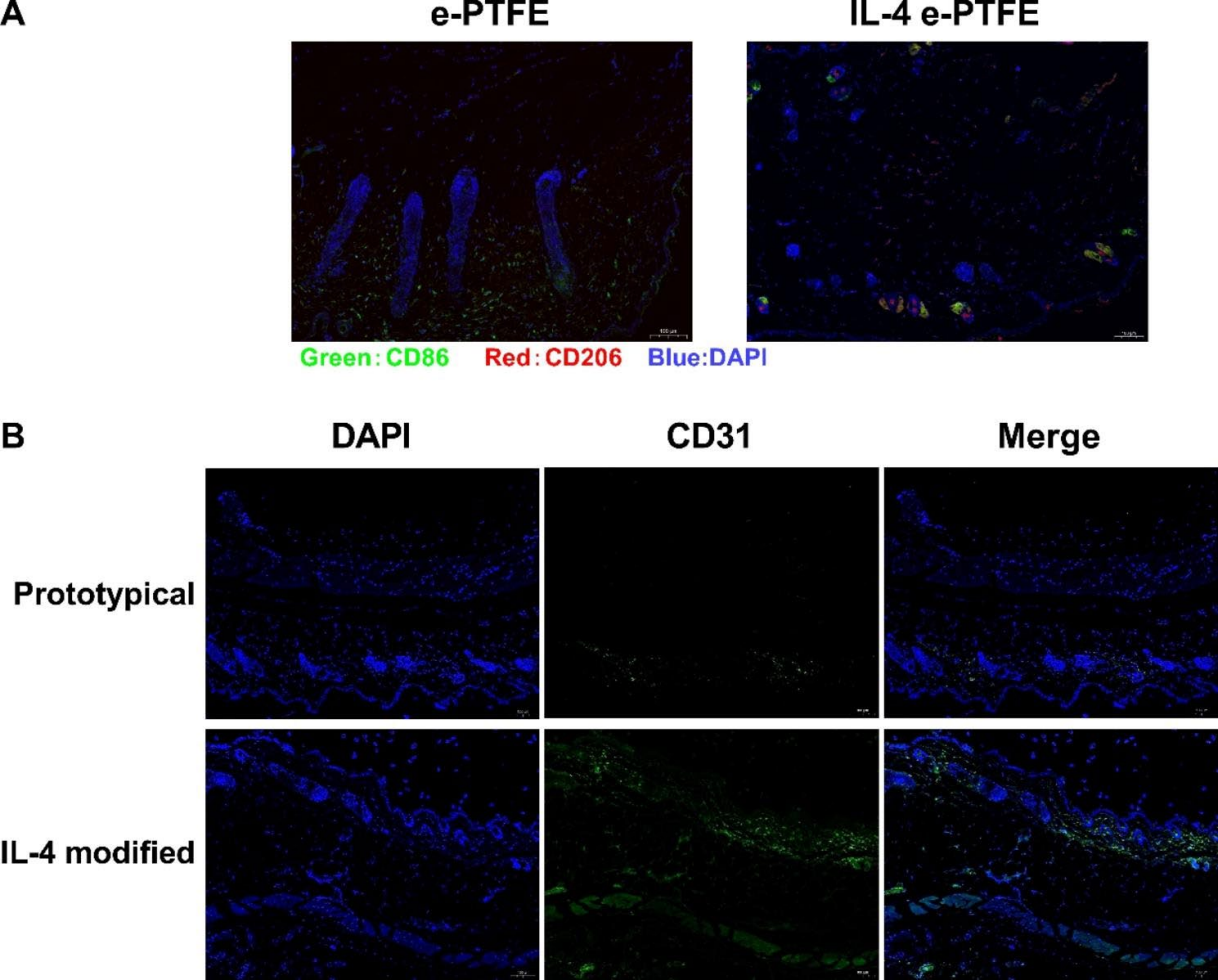
Detection of macrophages and vascular endothelial cell markers via immunofluorescence staining. **A**: Immunofluorescence staining results of macrophages; **B**: Immunofluorescence staining results of vascular endothelial cell markers

Immunofluorescence staining was employed to detect the levels of M1 macrophage marker CD86 and M2 macrophage marker CD206 in the skin flap transplantation area of rats in different models, and the results are as shown in Fig. 4A. The transplantation areas of rats in both the e-PTFE and IL4-e-PTFE groups had M1 macrophages, but M2 macrophages also existed in the skin flap transplantation area of rats in the IL4-e-PTFE group. In the meantime, as shown in Fig. 4B, the immunofluorescence intensity of vascular endothelial cell marker CD31 of rats in the IL4-e-PTFE was significantly higher than that in the e-PTFE group, which means the angiogenesis level of skin flap transplantation area of rats in the IL4-e-PTFE group was increased compared to the e-PTFE group. The above results prove that IL4-e-PTFE can promote angiogenesis of transplanted flap in vivo.

## Discussion

For most high polymer materials used in the physiological environment of human body, after contacting human body, some uncontrollable nonspecific proteins on material surface will be adsorbed, which will trigger a series of defense reactions of body, such as adverse reaction like platelet aggregation and inflammation of human body[21]. At present, one of the methods to improve the performance of biomaterial surface is modification of the material surface. For example, by using glucan as the substitute of polyethylene glycol, Massia et al[22]. fixed it to the surface of biomaterial via covalent bond, and the modified material surface can well exclude the nonspecific adsorption of protein. Later, on the basis of grafting glucan, they also grafted different polypeptides, and found that the biomaterial grafted with different polypeptides at the end showed selectivity in cell adhesion[22, 23]. IL-4 is a pleiotropic cytokine, which not has anti-inflammation effect, but can also promote angiogenesis. As for its application in biomaterial modification, there is only one report on the “immunoregulation” stent prepared based on IL-4 modified nanofiber regulation macrophage polarization[24]. In our study, after treatment with IL-4, the angiogenesis of HUVEC did not present much difference from the control group, but after co-treatment using IL-4 and monocyte, the angiogenesis level of HUVEC was significantly improved, and the expressions of the vascular endothelial cell markers CD31 and CD34 and the vascular endothelial growth factor (VEGF) PDEC-GF all increased. The above results prove that IL-4 has potentiation effect in HUVEC angiogenesis induced by monocyte. Moreover, in the cell experiment, with the extension of time in IL-4 treatment, the macrophages were gradually induced to transform to M2 macrophages, while under the treatment of M2 macrophages, the HUVEC angiogenesis increased. The above results prove that IL-4 can promote angiogenesis by inducing the formation of M2 macrophages.

The e-PTFE material has the polarization characteristics of low adsorption and hydrophobicity, and in recent years, related researches have been carried out to change its polarization to enable broader applications in the biomedical field. The e-PTFE material is one of the best candidate materials as the prosthesis for congenital diaphragmatic hernia (CDH) treatment, and even though this material has poor cell adsorption, its exposed surface in chest should present certain tissue adsorption. Via modification on one side of e-PTFE material, related researchers applied the polydopamine membrane formed on the water/air interface onto one surface of the e-PTFE membrane, so that it can face the enterocoelia surface to prevent tissue adhesion, while presenting certain tissue adsorption on the surface exposed in the chest, and the ultimate objective was to ensure that the e-PTFE prosthesis would still be favored in the congenital diaphragmatic hernia (CDH) surgical repair[1]. The e-PTFE artificial vascular graft has various great properties, such as easiness to suture, soft texture and compression resistance, but due to reasons such as thrombosis, application of these materials is restricted. Zhu et al.[25] utilized O-carboxymethyl chitosan to modify the surface of ePTFE vascular graft, which significantly improved the hydrophily of ePTFE, and the ePTFE blood vessel bound with O-carboxymethyl chitosan had great blood compatibility on surface, which increased the application of ePTFE artificial blood vessel in the medical field. In this study, by investigating the application of IL-4 modified e-PTFE in the skin transplantation process, we find that IL4-e-PTFE can not only inhibit apoptosis and inflammatory response in body, but also promote angiogenesis of skin flap, which has provided a reference method of using e-PTFE to reduce inflammatory response during skin transplantation process, and optimize the long-term effects of skin flap blood vessels.

## Electronic supplementary material

Below is the link to the electronic supplementary material.

Additional file: The images of original blots.

## Data Availability

All relevant data are available from the corresponding authors on request.
