# Supplementary material for: IL-4 modified expanded polytetrafluoroethylene (e-PTFE) surgical patch promotes angiogenesis in transplanted flap and inhibits inflammatory response
Source: BMC Surg. 2023 May 27;23:144. doi: 10.1186/s12893-023-02024-4 (PMC10225100; doi:10.1186/s12893-023-02024-4)
Supplement: Supplementary file 1 — Additional file: The images of original blots. [file 12893_2023_2024_MOESM1_ESM.docx]

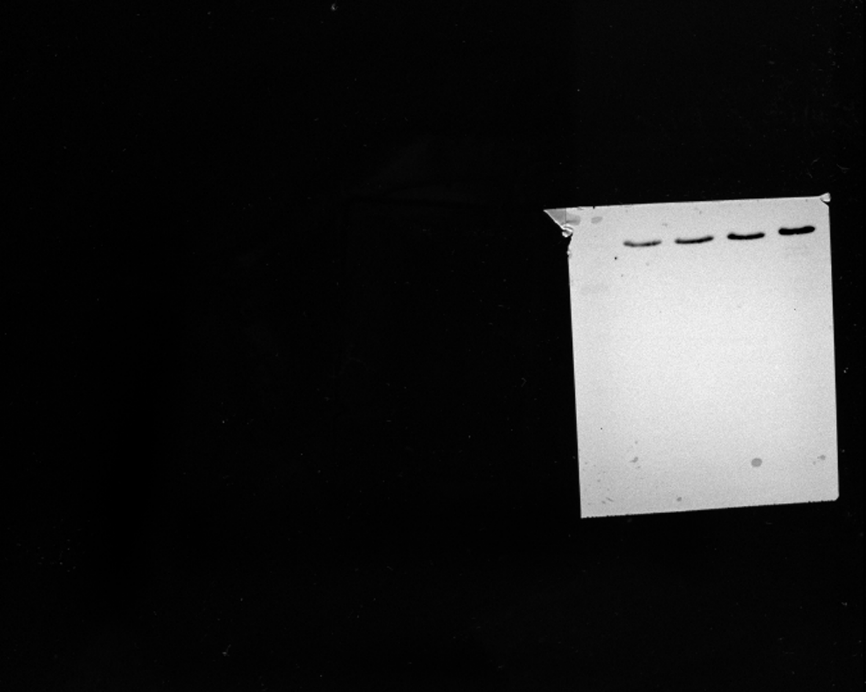


Fig 1B-CD31 original blots image.


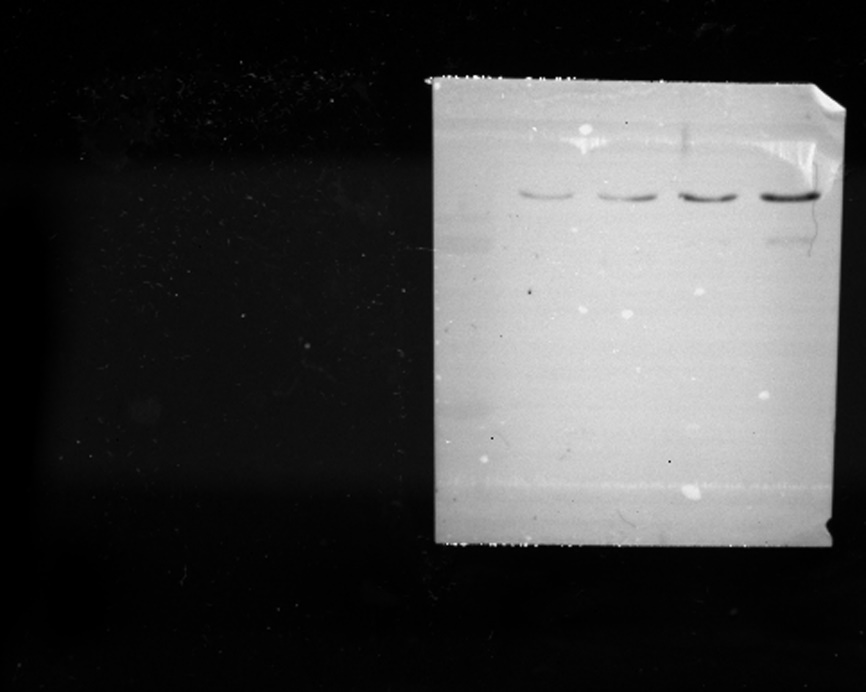


Fig 1B-CD34 original blots image.


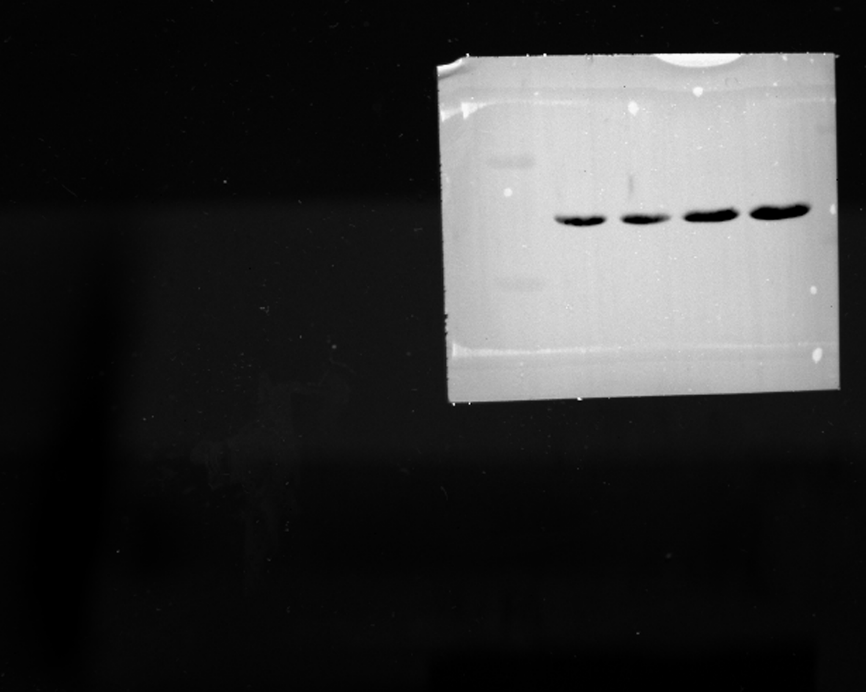


Fig 1B-PD-ECGF original blots image.


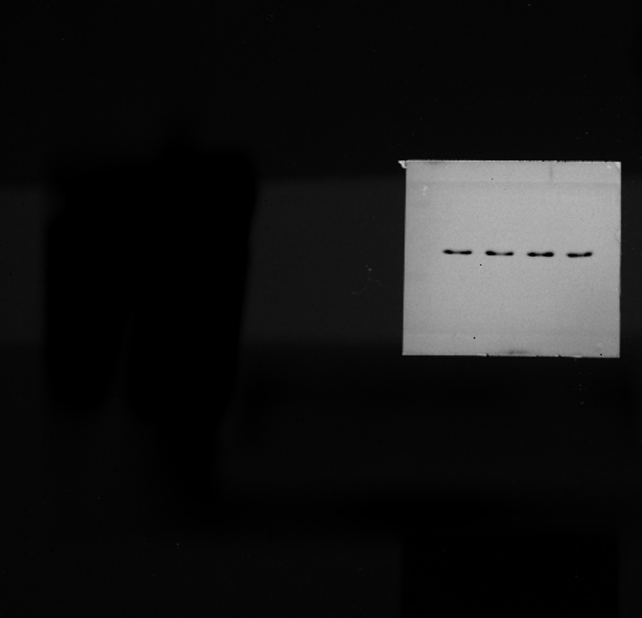


Fig 1B-Actin original blots image.


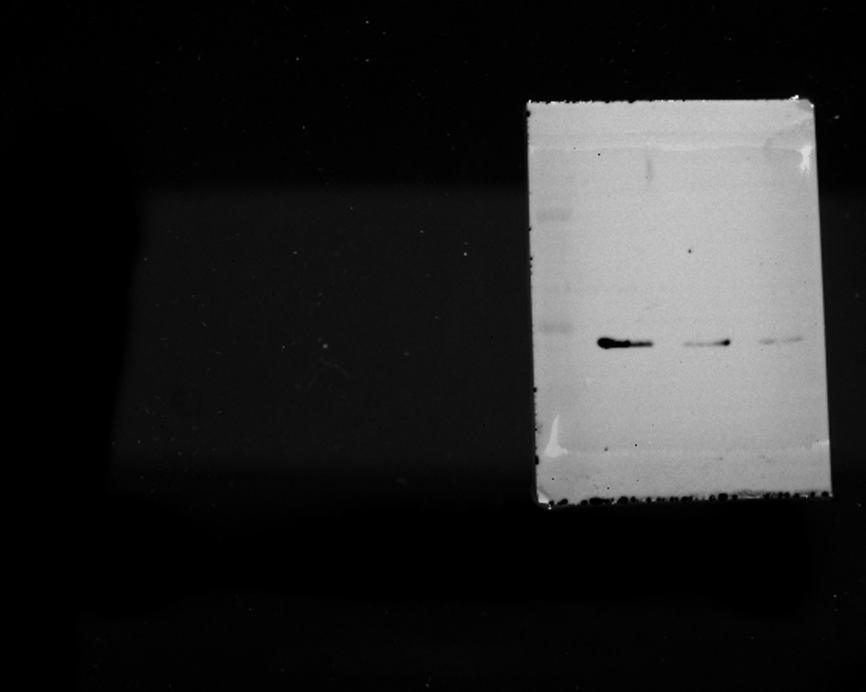


Fig 2A-IL-1B original blots image.


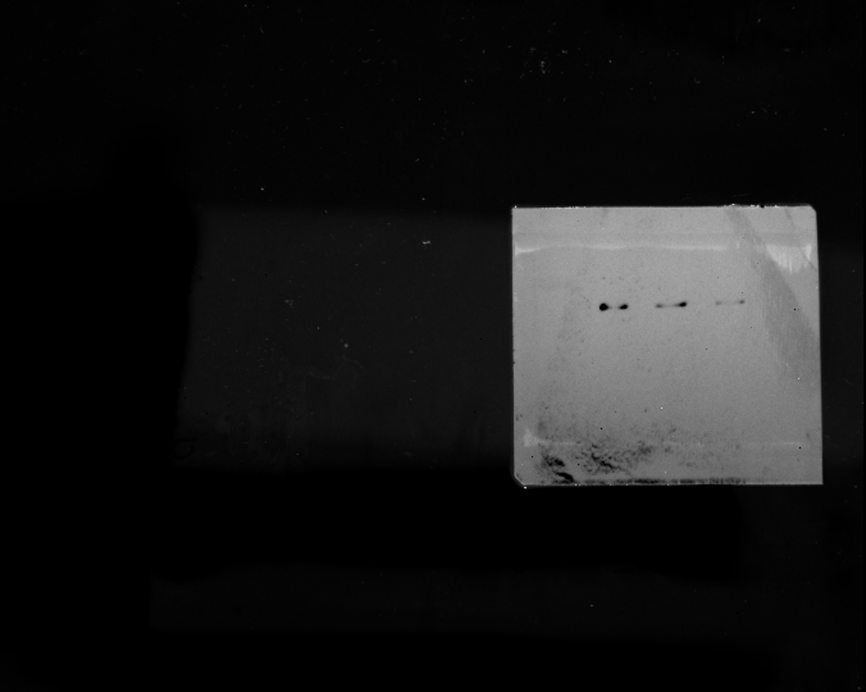


Fig 2A-CD86 original blots image.


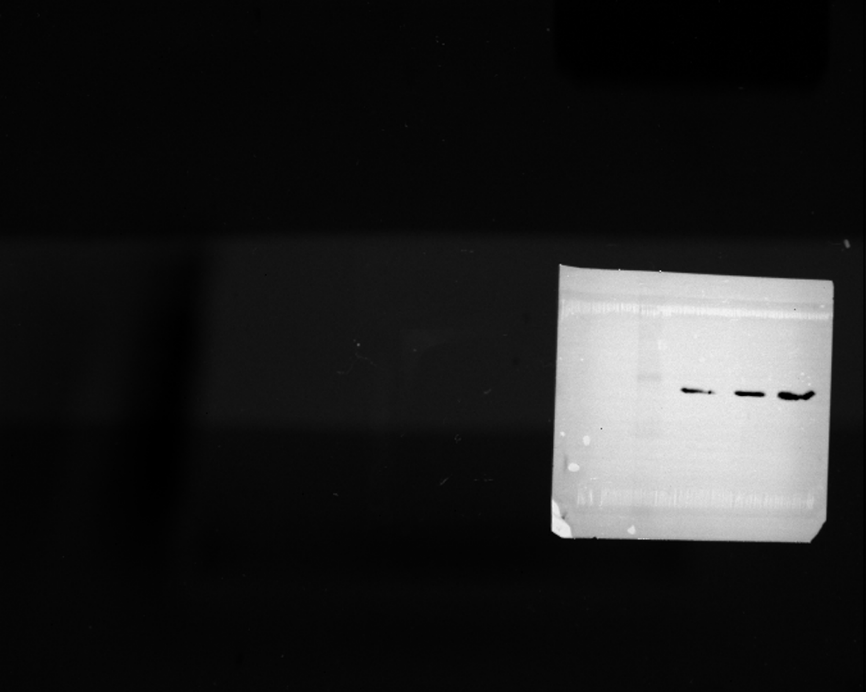


Fig 2A-ARG1 original blots image.


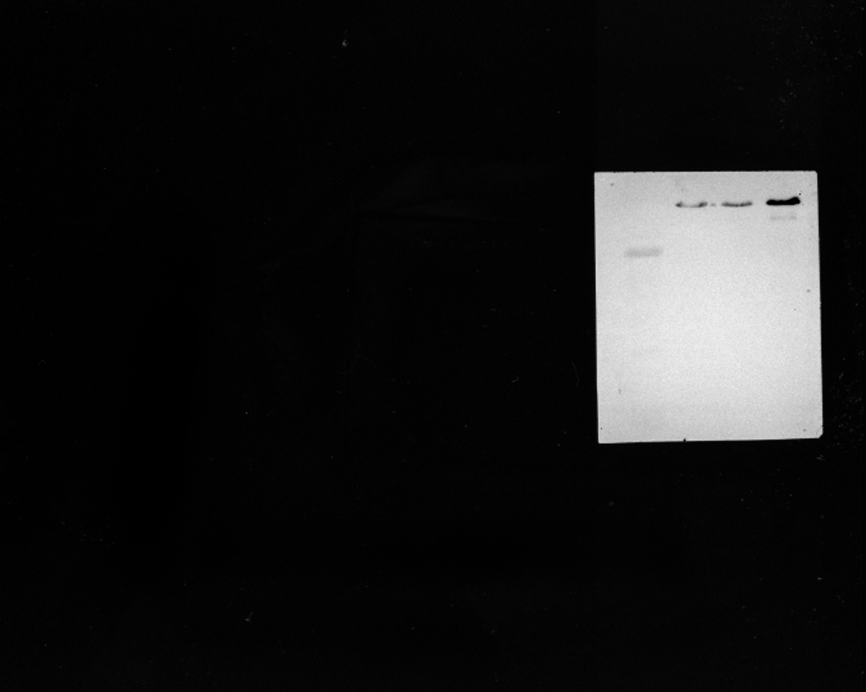


Fig 2A-CD163 original blots image.


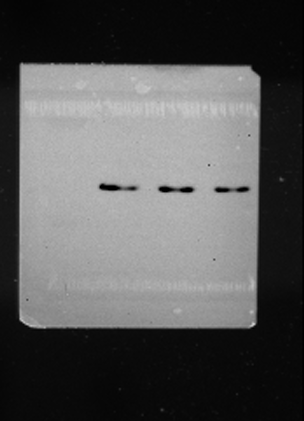


Fig 2A-Actin original blots image.


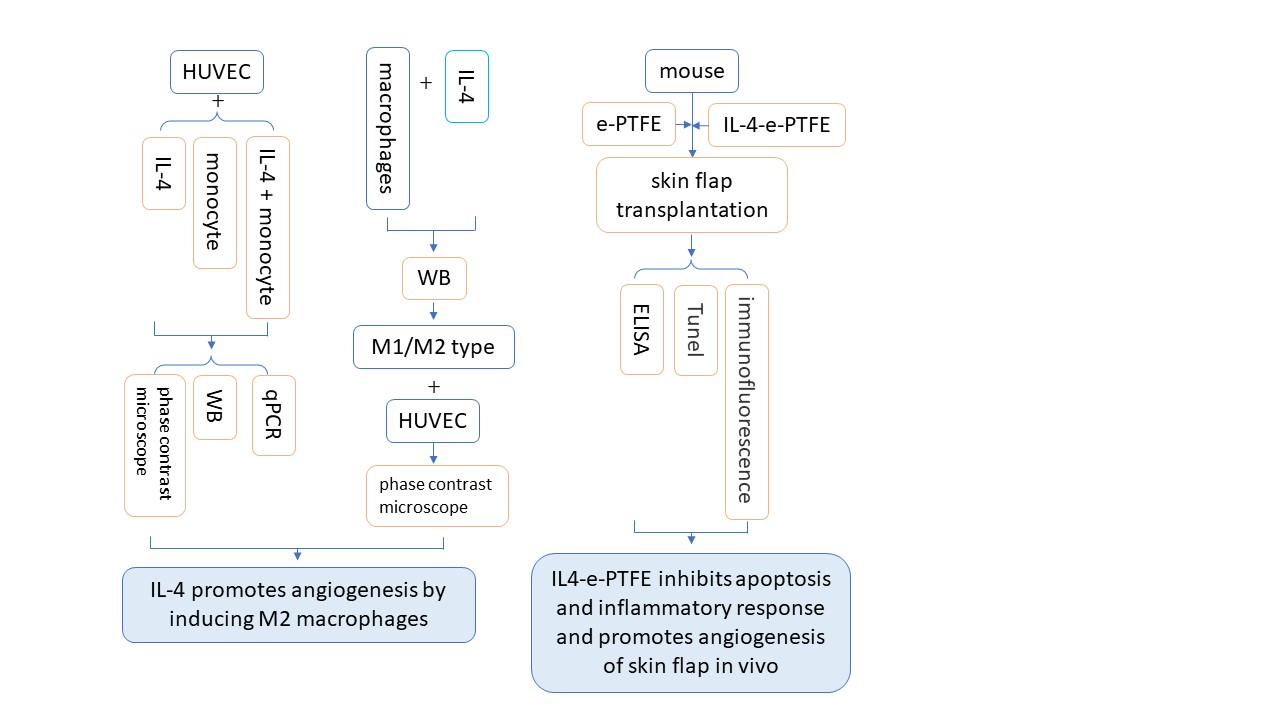


Supplementary figure 1. Process diagram.
